# First year doctors experience of work related wellbeing and implications for educational provision

**DOI:** 10.5116/ijme.5380.6ef1

**Published:** 2014-06-01

**Authors:** Helen M. Goodyear

**Affiliations:** Postgraduate Medical and Dental Education, Health Education, West Midlands, UK

**Keywords:** First year doctors, wellbeing, work related stress, educational supervision, career support

## Abstract

Objectives: To explore factors which affect newly qualified doctors’ wellbeing and look at the implications for educational provision.

**Methods:**

Data were collected by free association narrative interviews of nine Foundation doctors and analysed using a grounded theory approach. Two Foundation programme directors were interviewed to verify data validity.

**Results:**

Two main themes emerged: newly qualified doctors’ wellbeing is affected by 1) personal experience and 2) work related factors. They start work feeling unprepared by medical school, work experience (“shadowing”) or induction programmes at the beginning of the post. Senior colleague support and feedback are much valued but often lacking with little discussion of critical incidents and difficult issues. Challenges include sick patients, prescribing, patient/relative communication and no consistent team structure. Working shift patterns affects personal and social life. Enjoyment and reward come from helping patients, feelings of making a difference or teaching medical students.

**Conclusions:**

Whilst becoming familiar with their roles, newly qualified doctors search for identity and build up resilience. The support given during this process affects their wellbeing including coping with day to day challenges, whether posts are experienced as rewarding and how work influences their personal and social lives.

## Introduction

Health is defined as ‘a state of complete physical, mental and social wellbeing’.^1^ Work and wellbeing have a reciprocal relationship and are inextricably linked.^2^ Work related stress is highest in the first year of doctoring^3^ and decreases with seniority.^4^ Accepted dogma is that transition from medical student to first year doctor is notoriously difficult.^5^^,^^6^ Just the demand of starting work fulltime with a fixed start date, currently annually in the UK at the beginning of August is a stressor.^7^ Changes in medical school curricula and teaching and learning methods have helped prepare students for their first job, known in the UK as a Foundation Year 1 doctor (FY1D).^8^ However, medical school’s learning orientation is a solid scientific basis whereas FY1 is performance orientated and patients and medical staff expect new doctors to do a good job.^6^Restructuring of UK postgraduate training has occurred due to the Modernising Medical Careers (MMC) reforms.^9^ In addition, junior doctors’ “New Deal” regulations introduced shift patterns of work and weekly working hours were reduced to 48 in 2009 by the European Working Time Directive. Despite well-defined standards and close regulation of training by the General Medical Council (GMC), two independent inquiries recommended further improvements in Foundation Training.^10^^,^^11^There is a paucity of studies looking at first year doctors’ wellbeing particularly post-MMC as most recent studies look at preparedness rather than wellbeing. This qualitative study addresses the literature gap looking at factors that first year doctors’ feel affect their wellbeing and the implications for educational provision.

## Methods

The study was conducted in Health Education West Midlands (HEWM), UK. This is a large education provider looking after approximately 10% of the UK junior doctors.

### Study design

Free association narrative interviews were undertaken in combination with grounded theory methodology. An interpretive and relational paradigm was used and double hermeneutics (concomitant production of meaning and meaning-making) with both interviewee and interviewer contributing to the output.^12^ This paradigm views the world as complex and reality as subjective being constructed from people’s experiences and interactions. Personal identity emerges due a constant interplay between the environment and the subject’s inner world.Grounded theory draws on an emic (insider) understanding of the world with theory derived inductively from data rather than deductively from existing theory.^13^^,^^14^ There has been divergence in grounded theory methodology and this study followed Charmaz’s advice^14^ and read the literature prior to the study. An iterative approach with simultaneous data collection and analysis was used, comparing the findings from each interviewee before proceeding to the next interview.

### Sampling

Sampling was theoretical with the length of data collection and numbers of FYDs unknown at the start. Volunteers were sought at Foundation protected teaching sessions. After completion of the FYDs’ interviews, two Foundation Deans were interviewed who are senior Consultants responsible for the Foundation Programme. They were recruited by an e-mail asking for volunteers. Ethical approval was given by the Kent, Sussex and Surrey Education Department, UK (reference code MDM942012HG).

### Data collection

Over a 4 month time period, nine free association narrative interviews were undertaken using an open-ended question as recommended^15^: “Can you tell me how being a Foundation doctor has impacted on your life?” Follow up questions were open-ended aiming to achieve further narrative. All interviews were digitally recorded. A reflective diary and memos were kept to help with understanding of the data and so as to focus on key messages. After the data had been analysed it was discussed with the Foundation Deans.

### Data analysis

The free association narrative interviews and Foundation Dean interviews were transcribed verbatim by listening to recordings several times enabling immersion in the data and understanding of meaning, both obvious and hidden. Reflexivity was used to be aware of data manipulation and the influence I had on the data as well as strengths, weaknesses and interaction with the FYDs.^16^ Thematic content analysis of the data was carried out manually putting it into codes.^17^

### Validation

Analysis was done singly by the author as this work was part of a masters’ thesis. Data triangulation was achieved by collecting it from both FY1Ds and FY2Ds and after analysis discussion with the two Foundation Deans to ensure that during analysis and interpretation, meaning had been correctly understood. Data findings were used to construct theory.

## Results

Free association narrative interviews were undertaken with nine FYDs, two males and seven females. With the ninth interview, data saturation was achieved and no new insights were being gained relevant to the emerging theory.^14^ The majority of FYDs being female has been noted previously and suggests female trainees are happier to share their experiences. Three doctors were in FY1 and six in FY2. The 6 FY2 doctors had completed FY1 and could talk about their experiences in the whole FY1 year. Comments about FY2 were excluded from the data.The initial 77 codes were revised, summarised into 23 categories and six subthemes (Table 1). Two main themes were revealed which affect FY1Ds’ wellbeing: 1) personal experience and 2) work related factors. Overall, FY1 was considered as: “A very big culture shock” and “Learning to deal with not just your own life but having responsibility for other people as well”.

**Table 1:** Emergent themes and subthemes

Emergent Themes	Subthemes	Summary of codes
Personal experience	Feeling unprepared for the FY1 role	Medical student experience
		Induction
		Shadowing
		Confidence and competence
	Feeling FY1 affects personal and social life	Family support
		Friends/public support
		Social/personal life
		Affects life and career
		Doing things related to medicine in spare time
	Finding FY1 enjoyable and rewarding	Enjoyment and reward
Work related factors	Feeling (un)supported at work	Support from nurses
		Support from doctors
		Feedback
		Protected teaching
		Educational supervision
		Debriefing
		Team working
	Dealing with the challenge of being a FY1D	What the job entails
		Clinical challenges
		Hours of work
	Encountering difficult issues in FY1 training	Stressful situations
		On call issues

### Personal experience

The three key areas were feeling unprepared for the FY1 role, FY1 affects personal and social life and finding FY1 enjoyable and rewarding (see Table 2).

**Table 2:** Verbatim comments about personal experience of FY1Ds

Feeling unprepared for the FY1 role “Medical School is the Foundation of knowledge which you need’ but as for preparing you for your actual FY1 role, no”. “How to do a death certificate; that is one thing that medical school doesn’t teach you”. “I was told to go home [from induction] and come back for the night shift. I remember coming the first few weeks and everyone seemed to know everyone else’s names. I felt very lost, little things that I missed out on because I started on night”. “It took me three months to find where the toilet was”. “My first shift I was desperate for a drink of water after a few hours and I hadn’t stopped but I didn’t know where to get one from”.
Feeling FY1 affects personal and social life “Some of the on call rotas are not very good for sports. My team were not happy with me missing lots of training and lots of weekends”. “You are restricted in terms of certain weekends and nights. Often you leave late so things planned for that evening you may not be able to do”. “We do blocks of 12 days on call in a row; three or four long days, followed by three or four nights then three or four shorter days in a row – difficult switching your body clock back from night to days”. “I’ve taken a long time to learn the balance between work and taking things home --- It is knowing how to balance things, when to get exercise etc. ---My career choices have changed quite significantly. Looking at the female [medical] registrars--- coming in on their weekends, their days off --- It just made me sit back and think. You only get one chance to be here so I want to explore and experience everything that is out there rather than working all the time”.
Enjoying FY1 and finding it rewarding “The best thing is when you help somebody and they or their family are so grateful to you. The training and sitting down and reading the books which you have been doing for the last five years, you can actually put into practice and you feel useful”. “That’s [teaching medical students] the one thing that I actually did enjoy even on my first job”.


#### Feeling unprepared for the FY1 role

Medical school was felt to prepare FY1Ds for the knowledge aspects of work only. It was perceived as “a very controlled and safe environment; it doesn’t take into account, you have just worked the whole weekend. As a medical student you are sort of flitting on this and that ward not actually taking responsibility”.The number of practical procedures FY1Ds carried out as students varied between medical schools and type of rotation but opportunities were greatest in medicine and surgery. Four trainees had been to medical school outside the West Midlands. There was consensus that the undergraduate medical course could be better structured to make FY1Ds more prepared in the first few weeks of work. Medical student experience was also felt to be too dependent on what FYDs taught you which was very individual and on consultants who seemed remote figures.Shadowing practice, the process of observing and learning what is expected in role by being with the current FY1D varied considerably in duration and when undertaken. It ranged from a few days to six weeks and occurred months before to just prior to the FY1 post. Lack of shadowing guidelines was an additional factor: “I was quite keen to learn to write TTOs [to take out medications]. A lot of little things I could have picked up -- where the blood bottles are kept which are really helpful”.Shadowing was described as unhelpful if relocating to another region particularly if in a different specialty to the first FY1 post and as each hospital has a unique IT system. FY1Ds set up a further week’s voluntary shadowing for their second job if changing hospital or if the demands of the post were different e.g. moving from Paediatrics to Medicine.FY1Ds did not feel the induction programme was adequate with the main issue being lack of inclusion of day to day practicalities. As well as day to day clinical issues not being covered, induction also omitted vital information to cover basic physiological needs. Starting on nights was also problematic.

#### Feeling FY1 affects personal and social life

Sports and hobbies undertaken at medical school were either not possible during FY1 or limited by on call rotas and shift patterns of work. Several FYDs gave examples of working hours directly impacting on their social lives. Trainees felt a deep sense of responsibility and came to work despite ill health. There was a growing appreciation of a work life balance.The support of family, friends and a social network helped FY1Ds to cope with work challenges. Social life seemed difficult for those who had relocated with little contact with other doctors due to lack of organised events or a doctors’ mess which is a room with facilities for all junior doctors in the hospital to use for rest and relaxation. EPortfolio was disliked by all FYDs: “you are reflecting internally rather than formally documenting”.

#### Enjoying FY1 and finding it rewarding

Helping was a key theme for finding FY1 enjoyable, particularly related to patient care or teaching medical students.

### Work related factors

#### Feeling (un)supported at work

Medical consultants were around and came to the ward when called even if reluctantly whereas in surgery difficulties arose as consultants and senior trainees were usually in the operating theatre. Trainees described being desperate for help, often about medical problems in surgical cases (Table 3).

**Table 3:** Foundation doctors’ perceptions of support from senior colleagues, consultants and nursing staff

Positive “In my mind my seniors have been very supportive. It’s always nice to know that I have a safety net behind me that could step in if I felt of my depth at that point”, “When we first started, they [senior junior doctors] knew that we didn’t have a clue what we were doing”. “CDU [clinical day unit] was 1:1, you and the consultant [on the] ward round every morning five days a week and that was great”. “Knowing that if you were concerned or worried there was always someone there to call and seek advice. They were always very approachable”.
Negative “I was looking after 200 patients. I had one SHO [senior house officer] and one registrar”. “You would feel less supported [with the surgeons in theatre] whereas when the consultant is physically present just the fact that they can look at a patient always helps”. “It was a lot like old school medicine and now it’s not like that anymore unsupervised but actually it was. The consultant was like oh it’s a needle, stick it in and get some fluid out [of the chest]”. “Our consultant doesn’t come to the ward until 11 o’clock and only wants to see the new or sick patients” “Consultants did not see it [talking to bereaved relatives] as part of their role”. “I don’t think you get much support unless you are really screaming for it --- I remember practically crying down the phone [at this poor medical registrar] saying I just don’t know what to do, I really really need your help”. “I would just call the SHO in the next ward or somebody, anybody, whoever I could find”. “I have never been to a debrief. I cried, went home and was really upset actually, just went to sleep”. “I think it is one of those things that you have to pull yourself together, grab a quick cup of tea and carry on with what you are doing in the middle of the shift” [talking about what happens after a cardiac arrest]. “On the crash team, I just prayed that I was not the first person there. Eventually other people came. It wasn’t too long but it felt like forever”.
Dealing with the challenges of being a FY1D “I would say you are not a doctor, but a glorified secretary. You are doing paperwork so writing TTOs, discharge letters, referring, ordering bloods, ordering scans, interacting with the different professions - just a sort of go between”. “The admin side kept us behind at work. You would never hand over admin because no one was going to die. If you didn’t write the referral letter and didn’t get it done the next day, you would forget and the patient would never have the outpatient appointment”. “You end up doing things as you think it is probably my job. You do not have a sense of where your job ends, where someone else’s begins and where it overlaps -- you could never be sure what to expect from other team members”. “You are hoping that you are making the right decisions. Feedback is the one thing that has been the poorest managed. No-one tells you if you have managed a patient wrong or how you should do something”. “I called my mum and had a bit of a cry and I felt that got it sorted” [talking about a distressing incident]. “That support of I have had a rubbish day. Yes, me too I can understand and empathise with how you are feeling” [talking about sharing a house with other FYDs].

Nursing support was highly valued especially at the start of the job. However, not all nursing staff were supportive, removing cannulas at night after the recommended 72 hours maximum time in patients where it was very difficult if not impossible to get another one in. There seemed to be no sys-tem for doing these tasks during the day. Nursing staff could also push FY1Ds to work beyond their competence level especially in the first few weeks. Team working was highly valued but there was confusion as to who should do certain tasks. FY1Ds wanted feedback on their work which was often lacking.Educational supervision was a key area but one which “is so varied, some barely know your name”. All FYDs described clinical incidents in vivid and minute detail, which often involved young patients with severe disease or when treatment was futile despite over 12 months having elapsed for FY2Ds. After clinical incidents debriefing did not appear to take place.

#### Dealing with the challenges of being a FY1D

Patient administration was a role the extent of which FY1Ds had not expected especially the time it would take. They often felt “out of their depth” and lacking in the knowledge, experience and skills to undertake ward rounds on their own and to look after sick patients without senior support including one FYD managing a patient with an unstable airway for an entire night. FY1Ds talked of treatment trial and error and having to do something to help the patient. FY1Ds felt this excessive responsibility was a two-edged sword which helped them learn, in contrast to routine administrative tasks but as undertaken mainly at night was deeply anxiety provoking. Gaps on rotas exacerbated the problem of paucity of senior trainee support.Prescribing was another challenge for which FY1Ds did not feel well prepared: “I was petrified at prescribing. I think it is one of the biggest things that you do”. They felt confident with computer systems which did not allow incorrect doses and included blood test information in contrast to when this was not in place.Hours of work varied with both the type of post and between hospitals. A culture of working over one’s hours was not present in every post but was prevalent. FY1s tended to adopt a survival attitude to the year’s training. They felt that they just had to deal with all issues encountered so as to get through the year and be able to progress with their postgraduate training.

#### Encountering difficult issues in FY1 training

Communicating with patients and relatives and often breaking bad news was undertaken by FY1Ds. FY1Ds gave as much information as they could but acknowledged that some questions were beyond their knowledge. The main issue was the time it took, often getting behind with administrative tasks. Particular situations were likely to provoke anxiety e.g. cardiac arrests. The emotional aftermath was difficult to deal with especially if the FY1D had clerked in the patient. FY1Ds felt caught in the middle of consultant management disputes. They were expected to adopt new and often radically different management plans without questioning causing considerable distress.

### Foundation Deans’ (FDs) views

The two FDs agreed with the two themes and six subthemes of factors that affect FY1Ds’ wellbeing. Both agreed that

medical school, shadowing or induction did not prepare FY1Ds for their role and FY1Ds can find it difficult to establish a social network after relocating. They were aware that not all senior colleagues support FY1Ds and had incidents reported to them on Quality Assurance monitoring visits. They agreed that educational supervision was variable. FDs felt time management was a key skill which FY1Ds needed to learn especially prioritising results and being proactive and anticipating a patient’s needs. Both FDs acknowledged that this was difficult due to shift working patterns, FY1Ds being rushed off their feet and enforced lunchtimes for patients when emergency care only is undertaken. Staff shortages were acknowledged as a problem.

FDs did not agree with FYDs’ views on ePortfolio. They felt it was a training requirement, engagement was part of professionalism and better time management would make it less burdensome particularly if FY1Ds engaged with ePortfolio twice weekly.

## Discussion

The first year of postgraduate medical training is still perceived as challenging in agreement with previous reports.^5^^,^^6^ Despite a number of recommendations^18^ including two major reports^10^^,^^11^ there appears to have been little positive impact on UK first year doctors’ wellbeing. This work refutes suggestions that many challenges for first year doctors have been sorted. Some changes designed to improve wellbeing such as reduction in working hours, shift working patterns and hospital at night teams have brought their own stresses due to increased intensity and complexity of work. The feeling of lack of preparedness by medical school varies between schools as noted by Morrow et al.^19^ Shadowing practice also varied considerably in duration and when undertaken. Careful evaluation is needed of the mandatory four day paid shadowing period introduced in July 2012 and repeated in 2013.^20^Personal and social life was affected by shift working patterns, too little personal time due to long working hours and feelings of isolation in those who had relocated in agreement with previous studies.^21^^,^^22^ Family and friends were valued for their support but did not always understand what the job entailed. As found previously, informal peer support strategies are important.^22^ The negative view of ePortfolio supports findings that workplace based assessments are viewed as a tickbox exercise.^23^ FYDs gaining enjoyment from helping people or teaching students is one of four key motivators for applying to medical school.^24^ Lack of senior support was a major issue reflecting previous findings.^11^^,^^22^Educational supervision was much valued when in place but often lacking in keeping with the Collins report.^11^ This is despite clear GMC guidelines on supervision^25^ and wide availability of ‘Teaching the Teachers’ courses.

Due to financial pressures, there is a tendency to prioritise clinical work over educational roles such as supporting FY1Ds. The GMC (2012) implementation plan for trainer recognition and approval^25^ includes the importance of supporting and monitoring trainees’ educational progress, one of the seven standards of the Academy of Medical Educators.^26^ The situation of being desperate for help appears unchanged from studies over ten years ago.^18^ Interestingly these pleas challenge Scottish findings of FYD reluctance to call for help in acute care situations.^27^

Team working was highly valued. Confusion regarding team roles could explain why weakest communication skills with other team members has beenreported.^28^ The negative impact of lack of debriefing after clinical incidents is well described.^18^

FY1Ds taking excessive responsibility beyond their competence level coupled with paucity of senior support are long standing issues.^11^^,^^18^ As noted previously, acute care tasks are most likely to provoke anxiety.^29^ The importance of senior role models to show new doctors what is expected of them in their roles has been previously highlighted.^28^ Well described are the negative effects of being unfamiliar with day-to-day jobs and the ward environment.^5^ The challenge of prescribing is also well recognised.^29^ In contrast to previous studies,^18^ FY1Ds did not have communication difficulties with patients and relatives but found the time it took an issue due to getting behind with administrative tasks.

Limitations of this study include the work being undertaken in one educational provider area (HWEM) and participants were volunteers with the possibility that only those with strong views were sampled. However, four FYDs from medical school outside the West Midlands did not have different views regarding medical school preparedness. Common to all grounded theory studies is that author interpretation of data may represent the author’s own experiences and viewpoints although data triangulation by the FDs helped to safeguard against this possibility.

### 

#### Theory

**Figure 1:**
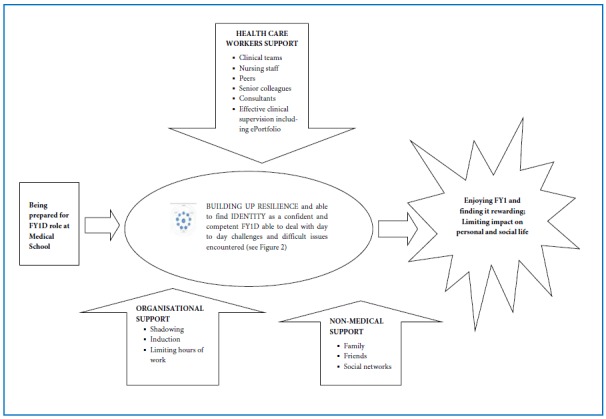
Support needed for first year doctors’ wellbeing

**Figure 2:**
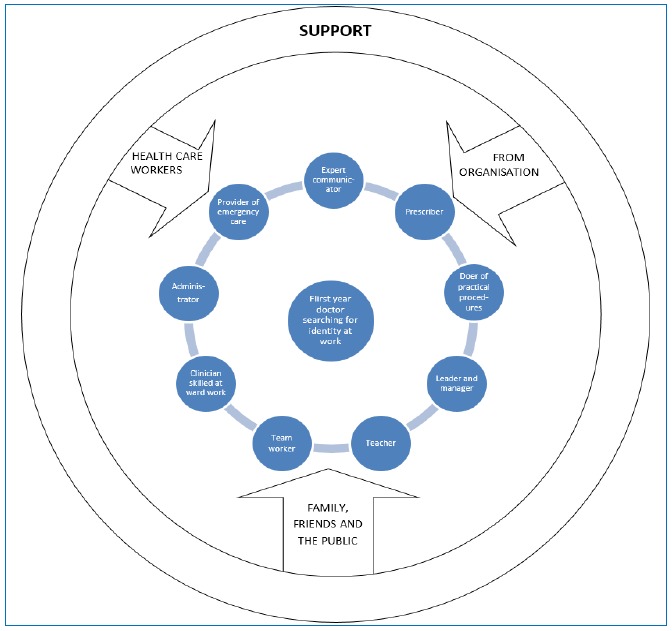
Support needed whilst first year doctors are searching for identity at work

Two key themes emerged from the data which affect FY1Ds’ wellbeing, namely personal experience and work related factors. Doctors vary in their past experience and personalities. It is important to focus on what can be changed which is support. This is essential for first year doctors to experience wellbeing at work and falls into four categories: 1) preparation by medical school; 2) health care workers support, especially senior colleagues and nursing staff; 3) organisational support and 4) non-medical support from family and friends (Figure 1). First year doctors have multiple roles at work (Figure 2), not all of which they experienced as medical students. Support shown in Figure 1 is essential for them to become familiar with their various roles (Figure 2). First year doctors develop these roles whilst simultaneously searching for identity and building up resilience, a dynamic capability allowing people to thrive on challenges, engage support and learn from difficulties.^30^ Resilience is greater when work has meaning in contrast to FY1Ds’survival attitude. Prior to and during this adjustment process support affects how first year doctors cope with day-to-day challenges, difficult issues encountered, whether posts are rewarding and the impact on personal and social lives.

## Conclusion

In conclusion, despite many UK postgraduate medical education changes, a number of factors need to be altered to improve first year doctors’ wellbeing. Provision of more support at work would positively affect their wellbeing. Currently support varies depending on type of post and hospital. Feedback, including for serious incidents, is often lacking. Educational supervision is also a spectrum. Responsibility for first year doctors varies widely from administration during the day to being in charge of seriously ill patients at night. Whilst becoming familiar with roles, first year doctors are searching for identity and building up resilience. The support given during this process is crucial.

## 

### Conflict of Interest

The author declares that she has no conflict of interest.
